# Impact of paranasal sinus invasion on advanced nasopharyngeal carcinoma treated with intensity‐modulated radiation therapy: the validity of advanced T stage of AJCC/UICC eighth edition staging system

**DOI:** 10.1002/cam4.1506

**Published:** 2018-05-01

**Authors:** Ying Wang, Jie Zhao, Yajie Zhao, Zhen Yang, Mingjun Lei, Zhanzhan Li, Rui Wei, Dengming Chen, Yuxiang He, Liangfang Shen

**Affiliations:** ^1^ Department of Oncology Xiangya Hospital Central South University No. 87, Xiangya Road Changsha Hunan Province 410008 China; ^2^ Department of Radiology Xiangya Hospital Central South University Changsha China; ^3^ Department of Nuclear Medicine Xiangya Hospital Central South University Changsha China

**Keywords:** Intensity‐modulated radiation therapy, nasopharyngeal carcinoma, paranasal sinus, prognosis, staging

## Abstract

The aim of this study was to clarify the prognostic role of paranasal sinus invasion in advanced NPC patients. Data of patients (*n* = 295) with advanced NPC (T3/T4N0‐3 M0) treated with intensity‐modulated radiation therapy were retrospectively analyzed. Staging was according to the AJCC/UICC eighth edition staging system. Overall survival (OS), local recurrence‐free survival (LRFS), distant metastasis‐free survival (DMFS), and disease‐free survival (DFS) were calculated, and differences were compared between patients with and without paranasal sinus invasion. Multivariate analysis was used to identify the independent predictors of different survival parameters. Paranasal sinus invasion was present in 126 of 295 (42.7%) patients. Sphenoid, ethmoid, maxillary, and frontal sinus involvements were present in 123 of 295 (41.7%), 95 of 295 (32.2%), 45 of 295 (15.3%), and 0 of 295 (0%), respectively. All survival parameters were significantly better in patients without paranasal sinus invasion. When paranasal sinus invasion was reclassified as T4 instead of T3, all survival rates, other than LRFS (*P *=* *0.156), were significantly better in the new T3 patients, and differences in all survival parameters remained nonsignificant between T3 with paranasal sinus invasion and T4 without paranasal sinus invasion patients (all *P *>* *0.05). In multivariate analysis, paranasal sinus invasion was found to be an independent negative prognostic factor for OS, DFS, and DMFS (*P *=* *0.016, *P *=* *0.004, and *P *=* *0.006, respectively), but not for LRFS (*P *=* *0.068). Paranasal sinus invasion has prognostic value in advanced NPC. It may be reasonable to classify paranasal sinus invasion as T4 stage.

## Introduction

Nasopharyngeal carcinoma (NPC) has assumed epidemic proportions in southern Asia. According to epidemiologic data from Globocan 2012 1, Asia and Africa account for 89.5% of all new cases reported from around the world, and China alone accounts for 38.2% of the total. Because of the location and pathological characteristics of NPC, treatment is mainly with radiotherapy. Intensity‐modulated radiation therapy (IMRT), which provides excellent local control and relatively better normal tissue protection, has gradually replaced two‐dimensional conventional radiation therapy and three‐dimensional conformal radiation therapy. A suitable staging system is indispensable for clinical decision making. Currently, two staging systems are widely used in China: the American Joint Committee for Cancer Staging (AJCC)/International Union against Cancer (UICC) eighth edition staging system and the Chinese 2008 staging system. The two systems differ mainly with respect to how paranasal sinus and masticator space involvement are used to classify the T stage (Table 1). Sze et al. 2 suggested that NPC with involvement of the medial pterygoid and/or lateral pterygoid muscle alone should be classified as T2 disease. Zhang et al. 3 proposed that parapharyngeal extension should be subclassified as mild or extensive and regarded as stages T2 and T4 disease, respectively. Pan et al. 4 recommended that medial pterygoid/lateral pterygoid involvement be classified as T2 instead of T4, and prevertebral muscle involvement also be included in T2 stage; the authors considered that this would lead to better distinction of hazards between different T categories. This recommendation was accepted by the AJCC/UICC eighth edition staging system for NPC. Various authors have recommended that, in addition to involvement of anatomical structures, tumor volume 5, 6, 7, distance between the primary tumor and the brain stem 8, and serum level of Epstein–Barr virus DNA 9, 10, 11 should also be considered as prognostic indicators for NPC patients. However, there has been little research on the significance of paranasal sinus invasion in the prognosis of NPC patients. The aim of this study was to clarify the impact of paranasal sinus invasion on prognosis of advanced NPC patients being treated with IMRT and to validate the AJCC/UICC eighth edition staging system.

**Table 1:** Comparison of T stage criteria in two staging systems for nasopharyngeal carcinoma

	The AJCC/UICC eighth edition staging system	The Chinese 2008 staging system
T‐primary tumor		
T1	Nasopharynx, oropharynx, nasal fossa	Tumor confined to nasopharynx
T2	Parapharyngeal extension, adjacent soft tissue involvement(medial pterygoid, lateral pterygoid, prevertebral muscles)	Nasal cavity, oropharynx, parapharyngeal extension
T3	Bony structure (skull base, cervical vertebra), paranasal sinuses	Skull base, medial pterygoid muscle extension
T4	Intracranial extension, cranial nerve, hypopharynx, orbit, extensive soft tissue involvement (beyond the lateral surface of the lateral pterygoid muscle, parotid gland)	Cranial nerve, paranasal sinus, masticatory space excluding medial pterygoid muscle, intracranial (cavernous, dural meninges)extension

## Methods

### Patients

The data of 295 patients with locally advanced NPC (T3/T4N0‐3M0) who received IMRT between August 2008 and December 2011 at Xiangya Hospital of Central South University (Changsha, Hunan Province, China) were retrospectively analyzed. All patients were diagnosed by nasopharyngeal biopsy and nasopharyngeal and neck MRI examinations. In addition to CT/MRI of the nasopharynx and neck, pretreatment workup included complete medical history, physical examination, chest radiography and/or chest CT (all patients with N3 disease underwent chest CT), B‐mode ultrasound scan of the abdomen and neck, bone scan, and blood hematology and biochemistry. Table 2 summarizes the characteristics of the patients. This study was approved by the Ethics Committee of Xiangya Hospital of Central South University (approval number 2011111087).

**Table 2:** Characteristics of the 295 locally advanced nasopharyngeal carcinoma patients

Characteristic	Variable	Paranasal sinus invasion		χ^2^	*P* value
		Yes (*n* = 126)	No (*n* = 169)		
Age (years)	<50	79	103	42.70	0.818
	≥50	47	66		
Gender	Male	91	120	0.05	0.819
	Female	35	49		
T stage (AJCC^a^)	T3	41	85	25.66	<0.001
	T4	85	84		
N stage (AJCC^a^)	N0	33	23	9.00	0.029
	N1	36	58		
	N2	42	56		
	N3	15	32		
Histology (WHO)	I	11	10	0.86	0.353
	II/III	115	159		
Chemotherapy^b^	A	6	8	2.78	0.733
	B	15	27		
	C	15	18		
	D	3	7		
	E	30	45		
	F	57	64		
Prescribed dose	<73.92 Gy	41	40	33.17	0.410
	≥73.92 Gy	85	129		

a According to the AJCC/UICC eighth edition staging system.

b A = no chemotherapy; B = concurrent or neoadjuvant or adjuvant chemotherapy; C = concurrent + neoadjuvant chemotherapy; D = concurrent + adjuvant chemotherapy; E = neoadjuvant + adjuvant chemotherapy; F = concurrent + neoadjuvant + adjuvant chemotherapy.

### MRI imaging, tumor volume measurement, and clinical staging

MRI was performed with a 1.5‐T Siemens Vision Plus scanner (Erlangen, Germany). The sequences included axial T1‐weighted imaging without fat saturation; axial T2‐weighted imaging; axial proton density imaging; sagittal T1‐weighted imaging; and postcontrast axial, coronal, and sagittal T1‐weighted imaging with fat saturation. The protocol has been described in a previous paper 5. The gross target volume of the primary tumor (GTV‐P) was calculated by the TPS using the summation‐of‐area technique, described in detail in our previous paper 5. The MR images for each patient were independently reviewed by two senior clinicians from the Departments of Radiology and Oncology, and all patients were staged according to the AJCC/UICC eighth edition staging system.

### Treatment

#### Radiotherapy

All patients received IMRT. Details of the radiotherapy technique have been reported previously 5. Briefly, the prescribed doses were 66.0–75.9 Gy to the planning target volume (PTV) of the GTV of the primary tumor (GTVnx); 66–72.6 Gy to the PTV of the GTV of metastatic lymph nodes (GTVnd); 59.4–64.0 Gy to the PTV of the high‐risk clinical target volume (CTV1); and 50.0–54.0 Gy to the PTV of the low‐risk clinical target volume (CTV2). The doses to the PTV of CTV2 were administered over 28 fractions, whereas all other doses were administered over 33 fractions. All patients were treated with simultaneous modulated accelerated radiotherapy once a day for 5 days a week. Dose limits for critical structures and plan evaluation were as defined by the Radiation Therapy Oncology Group (RTOG) 0225.

#### Chemotherapy

Chemotherapy was part of the treatment plan for all patients. Of the 295 patients, 281 of 295 (95.3%) accepted different forms of chemotherapy. Chemotherapy was not administered for 14 patients either because the patients refused chemotherapy or because they could not tolerate it. Neoadjuvant chemotherapy was administered when debulking of tumors was necessary or when the waiting time for radiotherapy was unacceptably long. After radiotherapy, adjuvant chemotherapy was administered to patients with N2/N3 stage disease and to those with evidence of residual disease on MRI or physical examination. Neoadjuvant chemotherapy or adjuvant chemotherapy consisted of 2–3 cycles of cisplatin plus 5‐fluorouracil or taxanes administered every 3 weeks. Concurrent chemotherapy consisted of cisplatin 80 mg/m^2^ every 3 weeks.

### Follow‐up

Follow‐up duration was measured from the first day of treatment to the date of last follow‐up or death. After radiotherapy, follow‐up examinations were conducted once every 3 months in the first 2 years, once every 6 months in years 2–5, and annually thereafter. Recurrence was defined as the tumor recurrence after disappearance for at least 1 month. The duration of overall survival (OS) was calculated from the day of radiotherapy completion to the date of death or last follow‐up. The duration of local relapse‐free survival (LRFS) was calculated from the day of radiotherapy completion to the date of detection of local recurrence of tumor. The duration of distant metastasis‐free survival (DMFS) was calculated from the day of radiotherapy completion to the date of detection of tumor metastasis. The duration of disease‐free survival (DFS) was calculated from the day of radiotherapy completion to the date of detection of tumor recurrence or distant metastasis, or death.

### Statistical analysis

All statistical analyses were performed using SPSS version 23.0 (IBM Corp., Armonk, NY, USA). Actuarial rates were calculated using the Kaplan–Meier method, and differences were compared using the log‐rank test. Multivariate analysis using the Cox proportional hazards model, with backward elimination of insignificant explanatory variables, was used to identify the independent predictors of survival. Statistical significance was set at *α *= 0.05. All *P*‐values were two‐sided.

## Results

### Treatment outcomes

**Figure 1:**
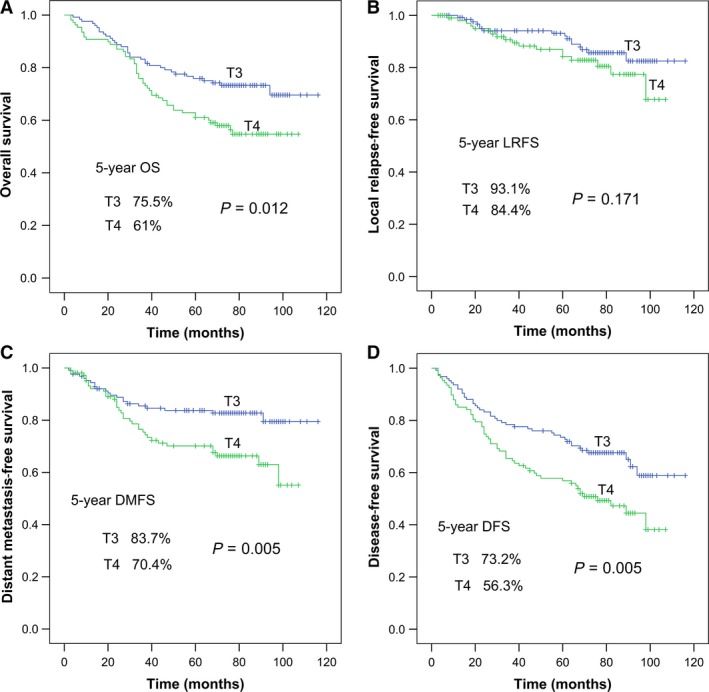
Survival curves of patients with NPC in the T3 and T4 subgroups (as classified by the AJCC/UICC eighth edition staging system): (A) the overall survival probability, (B) the local relapse‐free survival probability, (C) the distant metastasis‐free survival probability, and (D) the disease‐free survival probability.

Of the 295 patients included in this study, 60 were lost to follow‐up. Thus, the data of 235 patients were eligible for univariate and multivariate analyses. Median follow‐up was for 72 months (range, 3–116 months). In total, 30 of 235 (12.8%) patients developed local recurrence, 49 of 235 (20.9%) developed distant metastasis, and 12 of 235 (5.1%) developed local recurrence plus distant metastasis. There were 66 of 235 (28.1%) deaths, among which 45 were due to tumor recurrence and metastasis, 11 due to tumor‐associated complications, two due to nontumor factors such as stroke or gastrointestinal bleeding, and eighth due to unknown causes. Overall, the 5‐year OS was 68.8%, 5‐year LRFS was 89.3%, 5‐year DMFS was 77.8%, and 5‐year DFS was 65.4%. There were significant differences between the T3 and T4 subgroups in all survival rates except LRFS (Fig. 1): 5‐year OS was 75.5% vs. 61%, *P *=* *0.012; 5‐year LRFS was 93.1% vs. 84.4%, *P *=* *0.171; 5‐year DMFS was 83.7% vs. 70.4%, *P *=* *0.005; and 5‐year DFS was 73.2% vs. 56.3%, *P *=* *0.005.

### Paranasal sinus invasion

**Figure 2:**
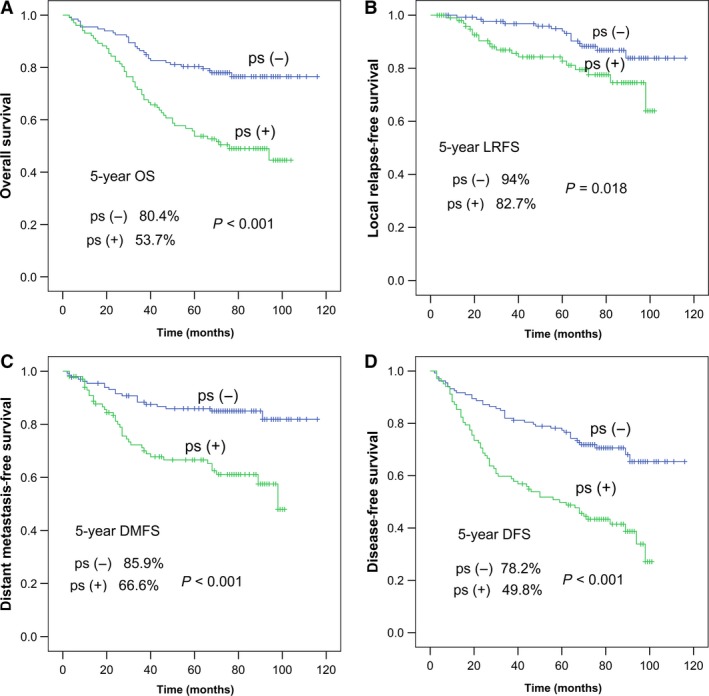
Survival curves of patients without paranasal sinus invasion (ps−) and with paranasal sinus invasion (ps+): (A) overall survival probability, (B) local relapse‐free survival probability, (C) distant metastasis‐free survival probability, and (D) disease‐free survival probability.

**Figure 3:**
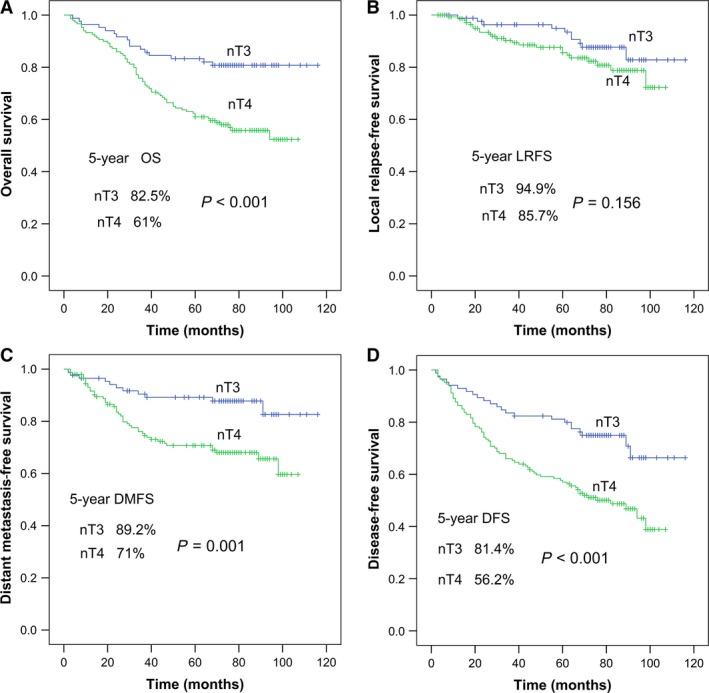
Survival curves of patients with nasopharyngeal carcinoma reclassified as nT3 and nT4 disease: (A) overall survival probability, (B) local relapse‐free survival probability, (C) distant metastasis‐free survival probability, and (D) disease‐free survival probability.

**Figure 4:**
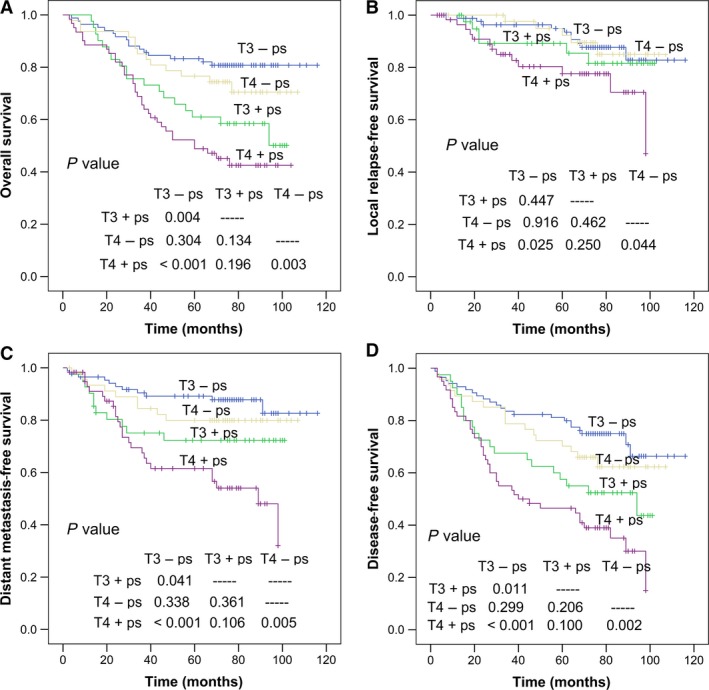
Survival curves of nasopharyngeal carcinoma patients with T3/T4 disease with and without paranasal sinus invasion: (A) overall survival probability, (B) local relapse‐free survival probability, (C) distant metastasis‐free survival probability, and (D) disease‐free survival probability.

Paranasal sinus invasion was identified in 126 of 295 (42.7%) patients. Among these 126 patients, the sphenoid sinus plus ethmoid sinus was involved in 48 of 126 (38.1%); the sphenoid sinus, maxillary sinus, and ethmoid sinus in 44 of 126 (34.9%); only the sphenoid sinus in 31 of 126 (24.6%); only the ethmoid sinus in two of 126 (1.6%); and the maxillary sinus plus ethmoid sinus in one of 126 (0.8%). The frontal sinus was not involved in any patient (Table 3). Survival rates were significantly better in patients without paranasal sinus invasion than in patients with paranasal sinus invasion: The 5‐year OS was 80.4% vs. 53.7%, *P *<* *0.001; 5‐year LRFS was 94.0% vs. 82.7%, *P *=* *0.018; 5‐year DMFS was 85.9% vs. 66.6%, *P *<* *0.001; and 5‐year DFS was 78.2% vs. 49.8%, *P *<* *0.001 (Fig. 2). We reclassified T3 patients with paranasal sinus involvement to T4 stage and termed them as nT4 and those without paranasal sinus involvement nT3, and then compared the survival of these newly created groups (Fig. 3). All survival rates, other than LRFS (*P *=* *0.156), were significantly better in nT3 stage patients. Next, we subdivided T3 and T4 patients into two groups each according to the presence or absence of paranasal sinus invasion; thus, T3−ps/T4−ps represented patients without paranasal sinus invasion, and T3 + ps/T4 + ps represented patients with paranasal sinus invasion. Among T4 patients, significant differences were observed in OS, LRFS, DMFS, and DFS between T4−ps and T4 + ps patients (all *P *<* *0.05; Fig. 4). Among T3 patients, significant differences were observed in OS, DMFS, and DFS between T3−ps and T3 + ps patients (*P *=* *0.004, *P *=* *0.041, and *P *=* *0.011, respectively); however, the difference in LRFS remained nonsignificant (*P *=* *0.447). Thus, those with paranasal sinus invasion were found to have worse survival, even within the same T stage. Meanwhile, nonsignificant differences were observed in OS, LRFS, DMFS, and DFS between T3 + ps and T4−ps patients (all *P *>* *0.05; Fig. 4).

**Table 3:** Distribution of paranasal sinus invasion in 295 locally advanced nasopharyngeal carcinoma patients by T classification

	Sphenoid sinus involved only	Ethmoid sinus involved only	Sphenoid sinus combined with ethmoid sinus	Maxillary sinus combined with ethmoid sinus	Sphenoid sinus, maxillary sinus, and ethmoid sinus	Total
T3	14	1	16	1	9	41
T4	17	1	32	0	35	85
Total	31	2	48	1	44	126

**Figure 5:**
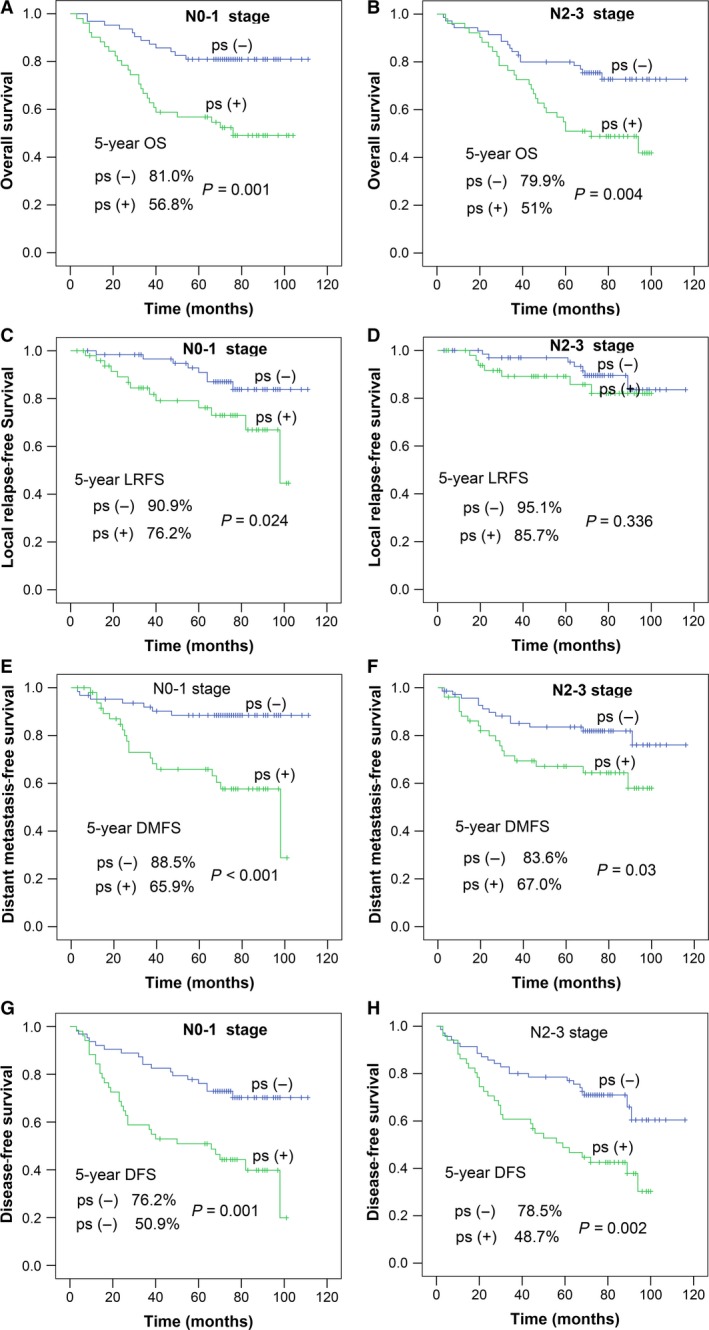
Survival curves of patients without paranasal sinus invasion (ps−) and with paranasal sinus invasion (ps+) in different N stages: (A, B) overall survival probability; (C, D) local relapse‐free survival probability; (E, F) distant metastasis‐free survival probability; and (G, H) disease‐free survival probability.

We further explored whether the effect of paranasal sinus invasion on survival was affected by N stage. For this, we evaluated the impact of paranasal sinus invasion on survival in patients with the same early N stage (N0–1) and late N stage (N2–3). We found that there was still a significant difference in all survival parameters within the same N stage; the only exception was LRFS in N2–3 stage patients (*P* = 0.336; Fig. 5).

### Multivariate analysis

Parameters that are generally considered to have significant effects on prognosis were included in the Cox proportional hazards model; these included age (<50 years vs. ≥50 years); gender (female vs. male); World Health Organization (WHO) histological grade (type II/III vs. type I); N stage (N0 vs. N1 vs. N2 vs. N3); use of chemotherapy and type of chemotherapy applied (no chemotherapy vs. concurrent or neoadjuvant or adjuvant chemotherapy vs. concurrent + neoadjuvant chemotherapy vs. concurrent + adjuvant chemotherapy vs. neoadjuvant + adjuvant chemotherapy vs. concurrent + neoadjuvant + adjuvant chemotherapy), primary tumor volume (GTV‐P; ≤46.4 mL vs. >46.4 mL) 5; prescribed radiation dose (>73.92 Gy vs. ≤73.92 Gy); paranasal sinus invasion (present vs. absent), invasion of bony structures (present vs. absent); and intracranial extension (present vs. absent). Paranasal sinus invasion was found to be an independent negative prognostic factor for OS (hazard ratio [HR] = 1.919; 95% confidence interval (CI): 1.128–3.264; *P *=* *0.016), DMFS (HR = 2.401; 95% CI: 1.283–4.492; *P *=* *0.006), and DFS (HR = 2.005; 95% CI: 1.249–3.217; *P *=* *0.004), but not for LRFS (HR = 1.910; 95% CI: 0.953–3.828; *P *=* *0.068). This suggests that paranasal sinus involvement is an independent negative prognostic factor for OS, DMFS, and DFS (Table 4).

**Table 4:** Multivariate analysis of advanced nasopharyngeal carcinoma patients

Endpoint	Variable	Regression coefficient	Standard error	*P*‐value	HR	95% CI	
						Lower	Upper
OS	Age	0.027	0.010	0.005	1.027	1.008	1.046
	Histology	−0.811	0.389	0.037	0.445	0.207	0.953
	N stage^a^	0.373	0.131	0.004	1.453	1.124	1.877
	Ps invasion	0.652	0.271	0.016	1.919	1.128	3.264
	GTV‐P	0.009	0.003	0.007	1.009	1.002	1.016
LRFS	Age	0.036	0.015	0.016	1.036	1.007	1.067
	Histology	−1.653	0.486	0.001	0.192	0.074	0.496
	Ps invasion^b^	0.647	0.355	0.068	1.910	0.953	3.828
DMFS	N stage^a^	0.445	0.156	0.004	1.561	1.149	2.120
	Ps invasion^b^	0.876	0.320	0.006	2.401	1.283	4.492
	GTV‐P	0.009	0.004	0.018	1.009	1.002	1.016
DFS	Age	0.018	0.009	0.032	1.018	1.002	1.036
	Histology	−0.725	0.347	0.037	0.484	0.245	0.957
	N stage^a^	0.392	0.117	0.001	1.480	1.177	1.862
	Ps invasion^b^	0.695	0.241	0.004	2.005	1.249	3.217
	GTV‐P	0.007	0.003	0.031	1.007	1.001	1.013

a AJCC eighth N stage.

b Ps invasion represents paranasal sinus invasion.

## Discussion

Advanced NPC can be difficult to treat because the tumor tends to be large and to metastasize. The therapeutic approach depends to a large extent on the clinical stage. In this era of IMRT, it is necessary to evaluate the rationality of the AJCC/UICC eighth staging system. At present, there is no consensus between the AJCC/UICC eighth staging system and the Chinese 2008 staging system stage with regard to how paranasal sinus invasion should be classified.

In this study, diagnosis of sinus involvement was based on MRI and simulated CT findings. Furthermore, every case of paranasal involvement was diagnosed by two senior clinicians from the Departments of Radiology and Oncology. MRI is useful for identifying extensive soft tissue invasion and distinguishing tumor from inflammation. On MR images, tumor usually presents as uneven thickening of the mucosa and a sinus cavity mass that is continuous with the primary tumor in the nasopharynx and has a similar enhancement signal. For recognizing damage to the continuity of the sinus wall, however, CT bone window is superior to MRI. Chong et al. 12 reported that CT could distinguish between inflammatory changes and tumor in the maxillary sinus, but was not helpful in the sphenoid and ethmoid sinuses. Chen et al. 13 found that MRI had good accuracy in diagnosis of T stage, whereas CT was better for identifying N stage and FDG PET/CT for M stage. Liao et al. 14 found significant differences between CT and MRI in ability to demonstrate invasion of the sphenoid sinus (CT, 13.6% vs. MRI, 16.7%; *P* = 0.029) and ethmoid sinus (CT, 7.1% vs. MRI, 3.3%; *P* = 0.004), but no significant difference between the two in ability to identify involvement of all paranasal sinuses (CT, 15.0% vs. MRI, 17.1%; *P* = 0.163) and maxillary sinus (CT, 2.4% vs. MRI, 2.6%; *P* = 1.000). In this study, we attempted to take advantage of the strengths of both CT and MR imaging.

In the present study, 42.7% patients had paranasal sinus invasion. Sphenoid sinus involvement was the most common (41.7%); ethmoid, maxillary, and frontal sinus involvements were seen in 32.2%, 15.3%, and 0%, respectively. The pattern was similar in the study by Liang et al. 15, where 242 of 943 NPC patients with stage I–IV disease had paranasal sinus invasion. Sphenoid sinus involvement was most common (163/943, 17.3%); ethmoid, maxillary, and frontal sinus involvements were seen in 50 of 943 (5.3%), 27 of 943 (2.6%), and two of 943 (0.2%), respectively. Tian et al. 16 retrospectively analyzed 770 patients with stage I–IV disease (by the AJCC seventh edition staging system). They reported paranasal sinus invasion in 182 of 770 (23.6%) patients, with invasion of the sphenoid, maxillary, and ethmoid sinuses being seen in 162 of 770 (21.0%), 86 of 770 (11.2%), and 38 of 770 (4.9%) patients, respectively. None of their patients had frontal sinus invasion.

In our review of the literature, we found only two papers that have discussed the classification of paranasal sinus involvement. Tian et al. 16 found that among patients with T3 disease, OS, DMFS, and LRFS were comparable for those with and without paranasal sinus invasion (*P *=* *0.22, *P *=* *0.15, and *P *=* *0.93, respectively). However, OS and LRFS were significantly better in T3 disease with paranasal sinus invasion than in T4 disease (*P *<* *0.01 and *P *=* *0.03, respectively). Therefore, the authors proposed that classification of paranasal sinus invasion as stage T3 was reasonable and that paranasal sinus invasion is an independent negative prognostic factor in NPC. However, it should be noted that the majority of their patients (80.2%) received two‐dimensional conventional radiation therapy; only 14.9% patients received IMRT. Another study has examined the same issue in patients receiving IMRT. Zhang et al. 17 retrospectively analyzed 1811 NPC patients. Paranasal sinus invasion was detected in 289 of 1811 (16.0%). Invasion of the sphenoid, ethmoid, and maxillary sinuses was observed in 271 of 1811 (15.0%), 89 of 1811 (4.9%), and 76 of 1811 (4.2%), respectively. No patient had frontal sinus invasion. Survival analysis showed that T3 patients with sphenoid sinus invasion alone had higher LRFS than those with ethmoid or maxillary sinus invasion (98.3% vs. 83.6%, *P* = 0.006), and T4 patients had similar LRFS as T3 patients with ethmoid sinus or maxillary sinus invasion (92.2% vs. 83.6%, *P* = 0.132). The authors therefore recommended that isolated sphenoid sinus invasion should be classified as T3 and ethmoid sinus and/or maxillary sinus involvement as T4. However, in their study, survival data were only obtained for 3 years and both Tian et al. and Zhang et al. used the AJCC/UICC seventh edition staging system and failed to take tumor volume, an important prognostic factor, into consideration.

Our survival analysis showed that LRFS was not significantly different between T3 and T4 stage patients. However when we stratified patients according to whether or not paranasal sinus invasion was present, we found significant differences in OS, LRFS, DMFS, and DFS. This suggests that paranasal sinus invasion is a predictor of poor prognosis in advanced NPC treated with IMRT. Meanwhile, no significant differences were observed in OS, LRFS, DMFS, and DFS between T3 + ps and T4−ps patients. Multivariate analysis confirmed that paranasal sinus invasion was an independent negative prognostic factor for OS, DMFS, and DFS. Thus, it is reasonable to recommend that paranasal sinus invasion should be classified as T4. However, paranasal sinus invasion showed no significant impact on LRFS in multivariate analysis or when T3 + ps and T3−ps patients were compared. This may have been because the 5‐year LRFS was high, and our sample was too small to detect a significant change.

We also found that significant differences in OS, DMFS, and DFS were present between patients with and without paranasal sinus invasion even within the same N stage (Fig. 5). Moreover, multifactor analysis—where the influence of N stage was adjusted for—ruled out the possibility that paranasal sinus invasion was only a confounding factor.

Some researchers have used nomograms to predict patient survival and compared the method with the current TNM staging system. Age 18, 19, 20, primary gross tumor volume 18, 19, 20, and invasion of ethmoid sinus 18, 19 should also be considered as independent prognostic factors.

Our results confirm that paranasal sinus invasion has prognostic significance in NPC, and we propose that patients with paranasal sinus invasion should be classified as T4 stage in the AJCC/UICC eighth edition staging system. There are two possible reasons for this difference between our findings and that of others. First, we used MRI and simulated CT as the diagnostic tool in our study. Others used CT, which may have led to some inflammatory lesions (arising due to blockage of the sinus) being misclassified as sinus invasion by tumor. Second, we included only locally advanced NPC cases in our study, and patients with paranasal sinus invasion had relatively large tumors.

This study has certain limitations. First, this was a retrospective study, and the treatments differed between patients. It is difficult to know whether the different chemotherapy regimens influenced the results of the study. Second, the sample size was small. The failure to identify paranasal sinus invasion as a significant prognostic factor for LRFS may have been due to the small sample size.

## Conclusions

Paranasal sinus invasion appears to be an important prognostic factor in NPC patients receiving IMRT. The results of this study suggest that in advanced NPC, paranasal sinus invasion should be classified as T4 stage in the AJCC/UICC eighth edition staging system.

## Conflict of Interest

The authors declare that they have no competing interests.
